# Size‐Related Variation in Tree Leaf Traits and Its Effects on Trait‐Growth Relationships in a Subtropical Cloud Forest

**DOI:** 10.1002/ece3.72169

**Published:** 2025-09-15

**Authors:** Yong‐Qiang Wang, Shi‐Dan Zhu, Han Wang, Kun‐Fang Cao, Hong‐Xiang Wang

**Affiliations:** ^1^ Guangxi Key Laboratory of Forest Ecology and Conservation, Key Laboratory of National Forestry and Grassland Administration on Cultivation of Fast‐Growing Timber in Central South China, College of Forestry Guangxi University Nanning China; ^2^ State Key Laboratory for Conservation and Utilization of Subtropical Agro‐Bioresources Guangxi University Nanning China; ^3^ Ministry of Education Key Laboratory for Earth System Modeling, Department of Earth System Science Tsinghua University Beijing China

**Keywords:** functional traits, intraspecific trait variation, montane cloud forests, size‐dependent effects, tree growth modeling

## Abstract

Trait‐based approaches offer an essential tool for exploring tree growth and adaptation strategies. However, the generality of trait‐growth relationships and the role of tree size in influencing their relationships remain uncertain. This study aims to explore size‐dependent trait variation and its effects on individual growth models using a trait‐based approach. We measured the leaf anatomical characteristics and nutrient content of 322 trees from 18 coexisting species and monitored their growth rates in a subtropical montane cloud forest. Our results showed that between 26% and 62% of trait variance was attributed to intraspecific variation of different sized trees. Larger trees tend to have smaller specific leaf area (SLA) and thicker palisade tissue (PT), while they also exhibit smaller and denser stomata to optimize water utilization and photosynthetic efficiency. As trees increased in size, their basal area growth advantage was attributed to both vertical competitive advantage and functional trait adaptations for light capture. Canopy species enhanced individual tree growth by adjusting the morphological structures of their leaves, such as thicker PT, higher stomatal density, and lower SLA, while understory species increased leaf phosphorus content, reflecting their specialized adaptation strategies to distinct vertical niches in phosphorus‐limited environments. In addition, traits measured at the individual level revealed broader trait‐growth relationships compared to species average traits. The study highlights that the pronounced effects of size‐dependent trait variation are crucial for elucidating trait‐growth relationships and understanding tree adaptive strategies under heterogeneous vertical light conditions.

## Graphical abstract

This study investigates how size‐dependent variation in functional traits influences individual tree growth in a subtropical montane cloud forest. By analyzing intraspecific differences in leaf anatomical and nutrient traits across 322 trees from 18 species, the findings reveal that larger trees exhibit lower SLA, thicker palisade tissue, and denser stomata, contributing to improved light capture, and that canopy and understory species adopt distinct trait strategies in response to vertical niche differentiation.

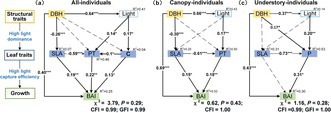

## 1 Introduction

The understanding of the underlying mechanisms of tree growth dynamics has remained a fundamental objective for ecologists and foresters, as tree growth is closely linked with forest productivity and carbon sequestration (Pommerening et al. 2020; Gourlet‐Fleury et al. 2023). In recent decades, trait‐based ecology has emerged as a novel framework for exploring tree growth strategies through functional trait adaptations (Bongers et al. 2020; Rubio et al. 2021). This approach emphasizes the significance of functional traits, defined as organismal characteristics that affect key plant functions, including resource acquisition (e.g., light capture), nutrient conservation, and defense against herbivory and abiotic stress (Laughlin et al. 2020; Chalmandrier et al. 2021). Although some studies show significant predictive relationships between traits and growth performance (Liu et al. 2016; Xu et al. 2023), accumulating evidence also suggests that tree growth is weakly associated with functional traits (Oktavia et al. 2022; Xu et al. 2023), and the consistency of their relationships may largely vary across biological organization levels and environmental gradients (Fajardo et al. 2024). Even with the growing interest of ecologists in trait‐based modeling, it remains unclear whether trait‐growth relationships are stable and to what extent the predictive power can be enhanced by integrating functional traits into conventional tree growth models in species‐rich natural forests.

Plant size is an important factor determining tree growth potential and often covaries with physiological traits across different stages of tree ontogeny. These traits, including photosynthetic, hydraulic, morphological, and biochemical properties, have been widely documented to exhibit considerable variation from seedlings to mature, emergent trees (Olson et al. 2018; Zheng et al. 2022). One recognized factor for the size‐trait covariance is the increased hydraulic path length with tree height, which may influence the structural development of leaf traits and branch wood density (Olson et al. 2018, 2020). Moreover, the heterogeneous environmental conditions across different forest layers, including variations in light availability and moisture, can largely influence trait variations of individuals for optimizing light and water acquisition (Bartholomew et al. 2022; Bin et al. 2022). Because individual trait variations closely reflect tree physiological processes and responses to environmental changes, recent studies suggest that individual trait measurements, compared to species‐average traits, provide deeper insights into the actual impacts of functional traits on tree growth and the intricate mechanisms behind tree performance (Yang et al. 2021; Kerr et al. 2022). Despite this focus, the link between individual traits and growth is still undetermined. A key factor is that the relationship between trait and growth depends on the overall phenotypic context that involves interactions among multiple traits as well as the influence of tree size (Gibert et al. 2016; Yang et al. 2021; Fajardo et al. 2024). For instance, the strong effects of tree size‐dependent trait expression can profoundly shape trait‐growth relationships and could potentially overshadow the predictive value of individual traits in growth models. This aspect has not been extensively discussed in the literature, yet it is crucial for fully understanding the impacts of individual trait features in growth models.

Species of different functional groups exhibit distinct functional adaptations to environmental stress, and their intraspecific trait–growth relationships can vary across species (Fajardo et al. 2024; Zhao et al. 2024). Due to the substantial heterogeneity in vertical microenvironmental conditions, these variations in functional adaptations are particularly pronounced between canopy and understory species within mature forests. For instance, the leaves of canopy species exposed to sunlight tend to be smaller and thicker, with higher nutrient concentrations per unit of leaf area (LA). This adjustment helps prevent overheating and water loss, while also ensuring a high photosynthetic capacity to leverage benign light conditions (Kenzo et al. 2015). In contrast, understory species, living in microhabitats with limited light, exhibit a more conservative strategy characterized by lower trait plasticity, which emphasizes efficient biomass allocation and survival. These understory species often have high specific leaf area (SLA) and have evolved distinctive strategies to maintain light compensation points below ambient light levels, ensuring adequate light absorption for photosynthesis (Long et al. 2011; Tooley et al. 2022). Due to their limited light exposure, small understory trees are particularly dependent on nutrient supply to sustain photosynthesis and growth (Li et al. 2018; Ye et al. 2023). For example, phosphorus addition has been shown to enhance photosynthetic performance in understory species or smaller individuals in phosphorus‐limited environments (Zhu et al. 2014; Liu et al. 2024). These findings underscore the contrasting patterns of individual‐level trait adaptation and plasticity between canopy and understory species, which are shaped by vertical habitat heterogeneity and reflected in their divergent phenotypic traits and nutrient‐use strategies.

Montane cloud forests are distinctive ecosystems found in the mid to upper elevation zones of mountains, typically with altitudes between 1000 and 3000 m. These forests are shaped by frequent dense fog, which alleviates hydraulic limitation but notably causes a decrease in light availability for plants (Cavelier et al. 1996; Dawson 1998). Compared to dry forest ecosystems, light availability is a more important limiting factor for tree growth in the montane cloud forests rather than water and nutrients (Fahey et al. 2016; Jiang et al. 2019). Consequently, trees in these forests have developed specific functional traits to live in low light environments, such as small leaves, low SLA, and high Rubisco content per unit LA (Eller et al. 2020). In a mature cloud forest, the spatially dominant trees benefit significantly from available light, while the understory individuals adapt to thrive in the filtered and dappled shadows with high SLA. As a result, leaf traits associated with photosynthesis, including LA, SLA, leaf anatomical structure, and nutrient composition, are expected to be crucial indicators of tree vitality and performance in these habitats. However, despite existing insights into trait adaptability in cloud forests, the comprehensive understanding of trait‐growth relationships and their size‐dependent effects remains largely underexplored.

In this study, we measured the leaf and branch traits of individual trees in a subtropical montane cloud forest and evaluated their potential as predictors for tree growth rates. Utilizing dendrometers for precise measurements of radial growth, we developed predictions of tree growth based on tree size, spatial structure dominance, and functional traits including SLA, wood density, leaf anatomy, and nutrient content characteristics. We aimed to investigate the size dependence of individual trait variation in this forest and to discern how the combined effects of tree size dominance and light‐capturing traits influence photosynthetic processes and predict tree growth. Our research was guided by two key hypotheses: (1) individual trait covaries with tree size to jointly influence physiological and morphological adaptations, and this size‐dependent trait variation can significantly shape trait‐growth relationships. (2) In the stratified montane cloud forest, size‐dependent light capture and growth strategies depend on the modulation of specific traits between canopy and understory layers, with canopy species modifying their morphological structures to optimize light absorption, while leaf nutrient content is important for understory species.

## 2 Materials and Methods

### 2.1 Study Site and Plant Materials

**FIGURE 1:**
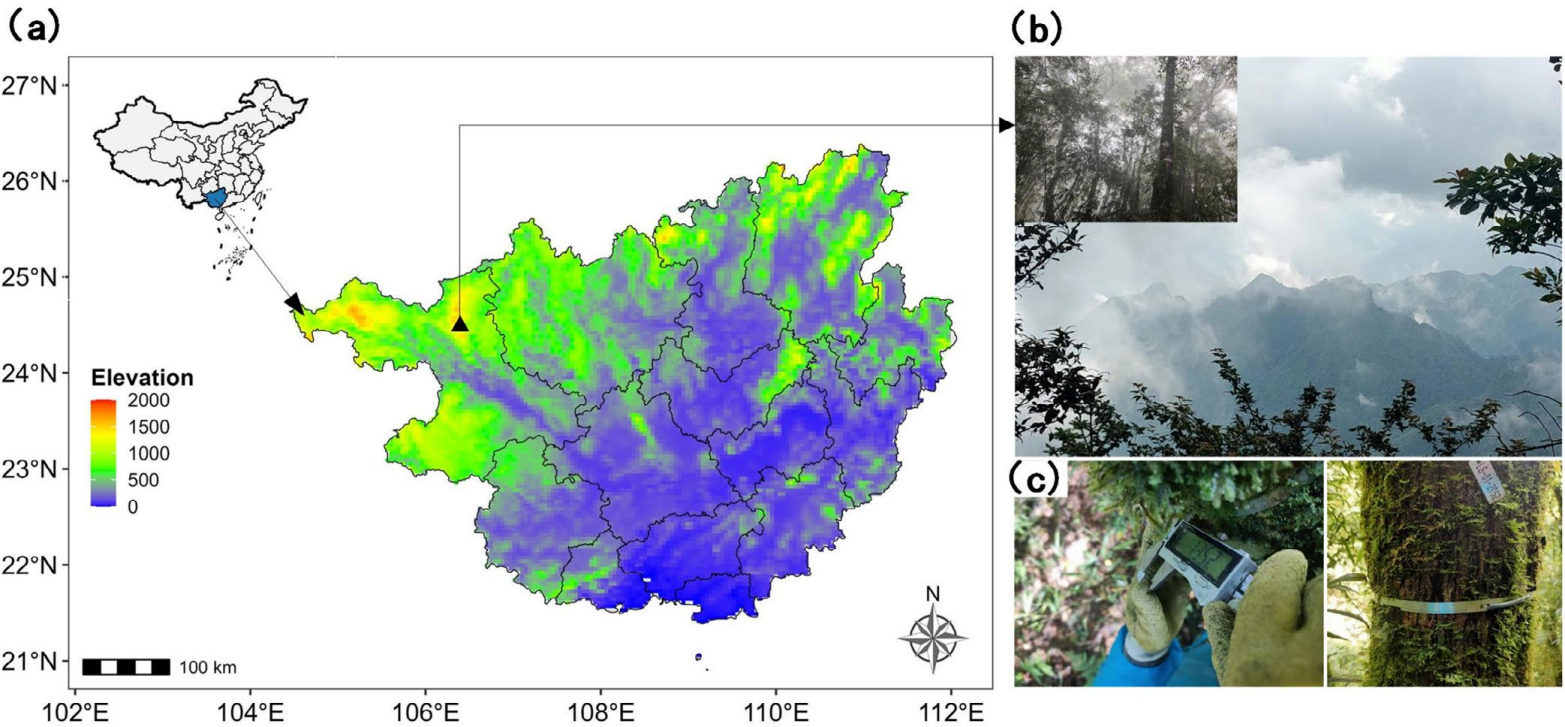
Map of China and Guangxi Zhuang Autonomous Region showing the location of the study site (left, a); Frequent heavy fog in its montane zone (top right, b); Tree growth increments were monitored by dendrometer bands, and measured with a digital caliper (bottom right, c).

The study was conducted at the Cenwanglaoshan National Nature Reserve in Tianlin County, Baise City, located within the Guangxi Zhuang Autonomous Region of China (Figure 1). This reserve covers an area of 25,212.8 ha (24°21′45″–24°32′7″ N, 106°15′13″–106°27′26″ E). The area is predominantly a subtropical montane cloud forest with elevations ranging from 1200 to 2000 m, featuring a mix of evergreen and deciduous broad‐leaved forest. The climate is subtropical and is typically influenced by monsoons. From 2015 to 2017, the average annual temperature was calculated as 13.7°C, with January having an average temperature of 4.7°C, indicating cooler conditions, while July showed warmer conditions with an average temperature of 20.7°C (Peng et al. 2019). The average annual precipitation was 1657.2 mm, with the rainy season lasting from May to October, accounting for 87% to 91% of the total annual rainfall (Peng et al. 2019). The forest is commonly characterized by frequent occurrences of fog and persistent low cloud cover, creating an ideal habitat for abundant epiphytes on trees (Song et al. 2015; Fahey et al. 2016).

In 2015, we established a permanent 100 m × 100 m forest monitoring plot in the Cenwanglaoshan National Nature Reserve. All woody plants with a diameter at breast height (DBH) greater than 1 cm were systematically tagged, numbered, positioned, and the species were identified and recorded. In 2018, we installed custom‐made stainless steel dendrometer bands with springs on sampled trees to monitor radial growth. Sampling focused on common and dominant species, primarily those with a DBH > 5 cm. Before installation, epiphytic mosses were removed from tree trunks, as they commonly cover stems in cloud forests (Figure 1c). In 2022, we re‐measured the forest plot and recorded the dendrometer readings. The established plot, situated at 1850 m and near the reserve's core zone, is frequently shrouded in clouds and mist (Figure 1), and thus represents typical conditions of cloud forest environments.

Trees from 18 common species were investigated based on their distribution characteristics within the plot, such as *Rhododendron simiarum* Hance, *Quercus jenseniana* Hand.‐Mazz., *Machilus leptophylla* Hand.‐Mazz., *Ilex formosana* Maxim., *Beilschmiedia fordii* Dunn., and *Camellia mairei* Melch. The selected species comprised six canopy species, six subcanopy species, and six understory species. Each of these species was represented by at least seven individuals in the plot, with the number of individuals per species ranging from 7 to 32. The general information for each species is presented in Table 1. A total of 322 individuals were selected and measured in the study.

**TABLE 1:** General characteristics of the 18 tree species studied.

Species	Number of individuals	Mean BAI and range (cm^2^ year^−1^)	Mean DBH and range (cm)	Canopy position
*Acer flabellatum* Rehde.	24	3.39 (0.39–13.16)	14.7 (7.2–29.7)	Canopy
*Castanopsis eyrei* Tutcher.	20	16.70 (0.62–59.90)	33.9 (8.3–61.8)	Canopy
*Quercus jenseniana* Hand.‐Mazz.	9	13.80 (0.93–24.46)	28.1 (8.2–54.9)	Canopy
*Dipentodon sinicus* Dunn.	31	6.36 (0.36–17.17)	14.1 (5.5–28.8)	Canopy
*Elaeocarpus japonicus* Siebold & Zucc.	18	15.32 (1.26–36.34)	26.9 (14.4–48.7)	Canopy
*Lithocarpus hancei* Rehder.	32	8.99 (0.07–32.37)	22.4 (5.0–57.7)	Canopy
*Beilschmiedia fordii* Dunn.	31	5.80 (0.06–49.41)	17.6 (4.7–46.2)	Subcanopy
*Clethra kaipoensis* H. Lév.	7	2.66 (0.68–7.92)	13.2 (7.5–29.5)	Subcanopy
*Machilus leptophylla* Hand.‐Mazz.	14	4.36 (0.03–15.29)	16.4 (5.3–29.2)	Subcanopy
*Symplocos lucida* Siebold & Zucc.	15	1.30 (0.04–3.75)	8.5 (5.2–16.0)	Subcanopy
*Schima argentea* E. Pritz.	11	5.86 (0.20–31.61)	15.2 (5.3–47.5)	Subcanopy
*Rhododendron simiarum* Hance.	22	3.95 (0.12–11.88)	15.1 (5.0–37.0)	Subcanopy
*Camellia mairei* Melch.	10	1.88 (0.34–8.18)	11.2 (6.4–22.3)	Understory
*Eurya impressinervis* Kobuski.	31	0.76 (0.10–2.75)	8.5 (5.1–15.1)	Understory
*Ilex formosana* Maxim.	15	1.74 (0.05–11.55)	7.5 (5.0–17.4)	Understory
*Neolitsea aurata* Koidz.	14	1.80 (0.11–8.77)	7.3 (5.0–15.0)	Understory
*Symplocos wikstroemiifolia* Hayata.	11	0.75 (0.04–2.75)	7.3 (5.4–9.9)	Understory
*Symplocos theophrastifolia* Siebold & Zucc.	7	3.24 (0.20–6.81)	12.3 (6.2–20.6)	Understory

*Note:* BAI is the average annual growth rate of the basal area (cm^2^ year^−1^), and DBH is the tree diameter at breast height.

### 2.2 Functional Trait Measurements

Functional trait measurements were conducted for each of the selected individuals. Two to three branches (150–200 cm long) were sampled from the crown for each tree using a telescopic pruner with a maximum length of 25 m. We selected small branches with mature and healthy leaves from sunlit branches, which were 40–50 cm in length. The small branches were carefully wrapped in black plastic bags to prevent light exposure and water loss, and their cut ends were inserted into a water bucket and subsequently transported to the laboratory. To minimize physiological changes, the branch samples were kept in darkness at room temperature for 12 h prior to analysis. We collected 20–30 leaves from each individual to measure leaf traits, such as SLA and leaf water content at saturation. Furthermore, we randomly selected three healthy and mature leaves from each individual tree for anatomical analysis. These selected leaves were then preserved in a solution of formalin‐acetic acid‐alcohol (FAA; 5 mL of 38% formalin, 5 mL of glacial acetic acid and 90 mL of 50% ethanol). In total, we collected 966 leaf samples for anatomy measurements from 322 individuals across 18 species (322 plant individuals × 3 leaves).

LA was measured with a LA meter (Li‐3000A; LiCor, Lincoln, NE, USA), and then the leaves were dried at 70°C in an oven for 72 h to obtain their dry mass. SLA (cm^2^ g^−1^) is calculated as the ratio of LA to leaf dry mass. To measure wood density (WD, g cm^−3^), a 5 cm segment was cut from each branch, split open with a knife to remove the pith and bark, and then the sample volume was calculated using the water displacement method (Perez‐Harguindeguy et al. 2016). The wood samples were dried at 70°C in an oven for 72 h to obtain their dry weight. WD was calculated by dividing the dry mass by the sample volume. Finally, the sapwood saturated water content (SWC_branch_, g g^−1^) was determined for the other half of the sample used in the WD calculation by dividing the water‐saturated mass by the dry weight of the sapwood.

During the measurement of leaf anatomical traits, leaf samples were first removed from the formalin‐acetic acid‐alcohol solution. Subsequently, the samples were prepared by cutting approximately 1 cm × 1 cm sections from both sides near the midvein of each leaf. We then used a sliding microtome (RM225; Leica Inc., Wetzlar, Germany) to obtain leaf cross‐sections measuring 8 to 10 μm in thickness. The sections were observed under a microscope (Leica DM2500, Germany) and randomly photographed as three pictures in a 0.23 mm^2^ microscopic field. The obtained images were measured using ImageJ 1.53 software to quantify the thickness of the upper cuticle (UCT), upper epidermis (UET), palisade tissue (PT), sponge tissue (ST), lower epidermis (LPT), and lower cuticle (LCT). Stomatal pore length (SPL), guard cell length (GCL), and stomatal density (SD) were measured using the nail polish imprint method. A uniform area of approximately 1 cm × 1 cm on the middle back of the leaf, excluding the midrib, was covered with nail polish. After 3 to 5 min, the nail polish was torn off with forceps to create a temporary mount on a microscope slide for subsequent observation and photography. Stomatal size (SPL and GCL) and density (SD) were measured in microscopic fields of 0.06 and 0.23 mm^2^ microscopic field, respectively.

For the measurements of leaf carbon (C), phosphorus (P), and nitrogen (N) content, leaf samples were first dried in an oven at 70°C for 72 h to a constant weight, and were ground using a mortar and pestle and sieved through a 65‐mesh screen. The leaf C content was determined using the potassium dichromate‐sulfuric acid external heating method. The N content was measured using the Kjeldahl method, and the P content was determined using the molybdenum antimony colorimetric method. Leaf C content was quantified as a percentage of the dry mass of the samples.

Finally, light exposure conditions of each individual were assessed using a visual crown illumination index described in previous studies (Verryckt et al. 2022). The illumination index ranges from 1 to 5, with the following categories: 1 represents complete shade with no direct light exposure; 2 represents low lateral light exposure; 3 corresponds to moderate vertical light exposure, where 10% to 90% of the vertical projection of the tree crown receives direct vertical light; 4 means that the tree crown is fully exposed to vertical light but lateral light is blocked within some or all of the 90° inverted cone encompassing the crown; and 5 signifies complete exposure of the crown to both vertical and lateral light (Verryckt et al. 2022). The summary characteristics of the measured traits for all sampled individuals are presented in Table 2.

**TABLE 2:** The means and coefficient of variation of the measured functional traits.

Functional traits	Abbreviation	Unit	Mean	Trait coefficient of variation (%)			
				All	Canopy	Sub‐canopy	Under‐story
Leaf area	LA	cm^2^	31.82	68.55	73.92	51.64	29.81
Specific leaf area	SLA	cm^2^ g^−1^	117.46	42.09	46.04	34.73	36.08
Leaf dry mass content	LDMC	g g^−1^	38.34	17.97	16.39	15.68	21.79
Leaf thickness	LT	μm	235.63	26.07	29.34	23.62	23.12
Upper cuticle thickness	UCT	μm	5.26	51.02	44.68	62.79	43.82
Upper epidermis thickness	UET	μm	19.54	41.93	38.80	41.93	28.46
Palisade tissue thickness	PT	μm	78.19	35.36	37.91	27.20	35.04
Sponge tissue thickness	ST	μm	115.08	33.02	34.53	30.47	26.14
Lower epidermis thickness	LET	μm	11.35	35.72	27.93	29.80	43.89
Lower cuticle thickness	LCT	μm	4.02	47.12	33.94	64.41	42.41
Stomatal density	SD	no mm^−2^	336.87	43.48	45.70	36.29	30.92
Guard cell length	GCL	μm	25.46	21.22	22.31	19.93	18.64
Stomatal pore length	SPL	μm	12.92	31.32	36.22	26.78	27.74
Leaf carbon content	C	%	37.87	7.52	5.81	7.63	8.35
Leaf phosphorus content	P	mg g^−1^	1.00	36.61	26.64	46.10	33.95
Leaf nitrogen content	N	mg g^−1^	13.20	20.53	16.75	21.84	22.62
Sapwood density	WD	g cm^−3^	0.54	12.84	14.06	12.58	11.16
Branch saturated water content	SWC_branch_	g g^−1^	0.51	9.87	10.51	9.95	8.06

### 2.3 Analysis

Tree growth rate is calculated as the average annual growth rate of the basal area increment (BAI, cm^3^/year) over 4 years. $\mathrm{BAI}=\pi \;\left({\mathrm{DBH}}_{1}^{2}-{\mathrm{DBH}}_{0}^{2}\right)/4$, where DBH represents the tree DBH, with DBH_1_ being the measurement in 2022 and DBH_0_ in 2018. The selection of BAI as the response variable in our tree growth models is informed by its capacity to more accurately reflect ability of trees to accumulate dry matter, alongside its proven robustness in tree growth modeling (Tenzin et al. 2017). BAI was later modeled based on the initial tree size, spatial competition, and functional traits. We used tree DBH as an indicator of individual size due to its close relationship with radial growth and its role in reflecting the tree's crown position in the vertical canopy structure, as demonstrated by the strong correlation between DBH and tree height (Figure S1).

The spatial explicit measures of tree competition were employed to represent the availability of light resources for each individual, as well as its dominance in size relative to its neighbors. Based on our initial analyses of various competition indices, we found that the hyperbolic tangent index, which is used to assess tree spatial dominance index (SDI), proved to be a better predictor of growth rates compared to other indices such as the Hegyi index as well as its derived indices. Pommerening et al. (2020) defined it as

$${\mathrm{SDI}}_{i}=\frac{1}{\sum w_{j}}\sum_{j=1}^{n}\frac{d_{i}^{2\alpha }}{d_{i}^{2\alpha }+d_{j}^{2\alpha }}\times w_{j}$$

(1) $$w_{j}=\frac{g_{j}}{\mathrm{dis}t_{j}^{\delta }}$$

where ${\mathrm{SDI}}_{i}$ is the hyperbolic tangent index quantifying tree spatial dominance and the level of exposure to light. $d_{i}$ and $d_{j}$ denote the sizes of a reference tree i and its nearest neighbor j, respectively. *n* is the number of nearest neighbors in the vicinity of a reference tree. Here, we define trees located within a radius of 5 m around a target tree as interaction competitors, as most of the tree crown radius were less than 5 m. The parameter *α* introduces ecological symmetry or asymmetry competition in the model. *α* = 0 indicates symmetric competition where limited resources are equally distributed among trees. In contrast, *α* → ∞ indicates the asymmetric competition situation where larger trees have disproportionate advantage in reaching resources over smaller trees. The weight $w_{j}$ models how the dominance of neighbors diminishes with increasing distance, and a greater weight was assigned to closer and larger neighbors. It is a function of the basal area of tree *j* and the Euclidean distance between trees *i* and *j. δ* is a parameter that emphasizes the influence of closer neighboring trees located in the vicinity of a subject tree *i*. In this study, we set *α* to 1 to indicate a moderate level of asymmetric competition, and *δ* is recommended to be chosen as 2 implying Gaussian competition kernel functions (Pommerening et al. 2020, 2023).

We initially examined the relationships between tree growth and the above‐measured traits. Then, hierarchical linear regression models (HLM) were used to predict tree growth from tree size, spatial dominance, and functional traits. Since the variation in growth rates between species accounts for a substantial proportion of our dataset, tree species identity was considered as a random effect that affects both the model intercepts and the slope of initial tree size, acknowledging that each species exhibits a unique growth pattern and responses to size. We first constructed a basic model that only used tree size and spatial dominance as the explanatory variables to BAI. Stepwise regression was then applied by adding functional traits to determine the most important variables and to establish the optimal model for individual growth rates. The response variable BAI was log‐transformed in order to increase the normality of the prediction residuals. Variance Inflation Factor (VIF) was calculated to assess the presence of multicollinearity among the predictor variables in each model, with variables retained if their VIF values were below 5. Different regression models were compared based on their Akaike information criterion (AIC) and were considered equal if the difference in AIC was less than two units. We also calculated marginal *R* ^2^ (the variance explained by fixed factors alone) and conditional *R* ^2^ (the variance explained by both fixed and random factors) for each model to identify the role of fixed and random effects. To explore if trait‐growth relationships can be strengthened by using individual‐level traits, regression models were constructed by using both individual traits and species average trait values. Species‐level trait values were used to interpret the intercept or slope in the basic model. Species maximum height (*H*_max_) was also used as a trait variable in the species‐level trait regression models. Considering that the studied forest is an old‐growth forest where the species adult stature is stable, *H*_max_ values were determined by the average of the five largest trees of each species within the established plots (five hectares in total). Finally, a relative importance analysis (RIA) was employed to examine the contribution of different explaining predictors to the model and to understand the sensitivity of growth to functional traits.

We used structural equation modeling (SEM) to explore the causal relationship among tree size, light, functional traits, and growth, and to see if any trait interactions affect growth. Initially, 10 traits were included in the SEM model based on their fundamental physiological meanings on tree photosynthesis. These traits included PT, SD, SPL, LA, SLA, leaf dry matter content (LDMC), leaf thickness (LT), as well as leaf carbon (C), nitrogen (N), and phosphorus (P) contents. In the null model, we assume that these leaf traits are directly linked to controlling growth rate while also being dependent on individual tree size and light conditions. This indicates that, in addition to the direct effect of size and light on the growth rate, they may also have an indirect path to tree growth via their impact on functional traits (Rowland et al. 2021). Additionally, traits may indirectly affect growth via other traits. To validate the null model and simplify it, we then performed a stepwise removal of the non‐significant paths. Model fit was assessed using various model comparison criteria, including the AIC, likelihood ratio chi‐square (*χ* ^2^), and comparative fit index (CFI). All the above analyses were performed in R v4.2.3 statistical software (R Core Team 2023), with packages “nlme v3.1‐168” “ggplot2 v3.5.2” “Lavaan v0.6‐19” and “relaimpo v2.2‐7” used for data visualization and regression analysis.

## 3 Results

### 3.1 Functional Trait Variation and Its Associations With DBH Sizes

**FIGURE 2:**
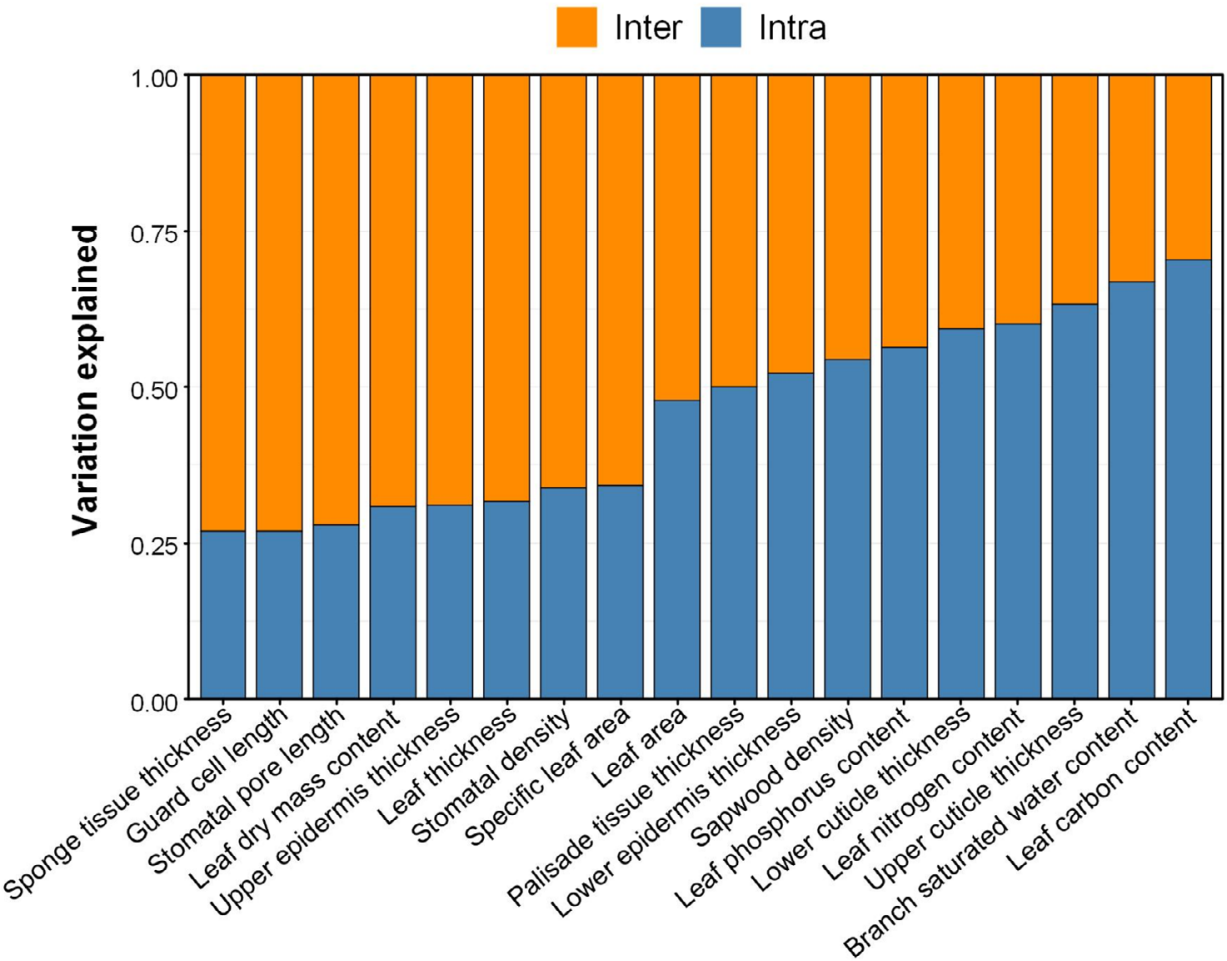
Variance in trait values explained by interspecific (orange) and intraspecific variation (light blue).

The coefficient of variation (CV) for the 18 functional traits ranged from 7.52% (leaf carbon content) to 68.55% (LA), indicating considerable trait variations among individuals (Table 2). ANOVA analysis revealed that between 26% and 62% of trait variations were explained by within‐species factors (Figure 2). In contrast to leaf traits, two branch traits, SWC_branch_ and WD, exhibited relatively low variability, with overall CVs of 9.87% and 12.58%, respectively. The correlation matrix (Figure S2) showed significant positive correlations among LT, LDMC, PT length, and UET thickness, whereas SLA was negatively correlated with these traits. Additionally, SD exhibited highly negative correlations with GCL and SPL, with correlation coefficients of −0.49 and −0.64, respectively (Figure S2).

**FIGURE 3:**
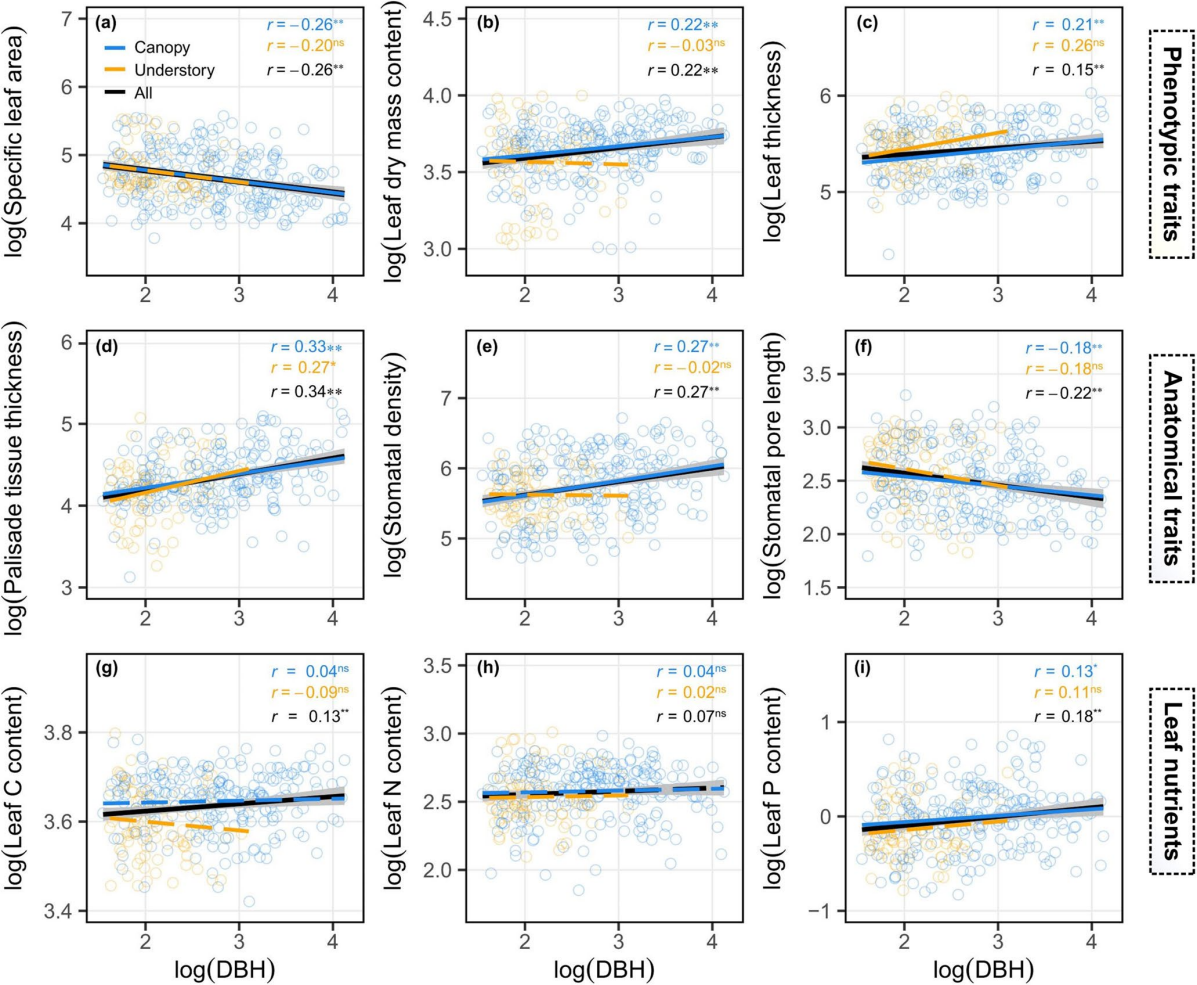
Relationships between log‐transformed leaf traits and diameter at breast height (DBH) across canopy and understory species, revealing significant size‐dependent variation across multiple types of traits. (a–c) Phenotypic traits; (d–f) Anatomical traits; (g–i) Leaf nutrients. Solid lines highlight significant regressions, and dashed lines show non‐significant relationships. Line colors denote different tree species categories: Light blue for canopy tree species (including both canopy and subcanopy species), orange for understory tree species, and black for all individuals combined. Gray shading represents the 95% confidence interval to the fitted regression line based on all data and *r* values indicate the correlation coefficient and the significance level of the DBH‐trait relationships. Asterisks denote statistical significance: *p < 0.05, **p < 0.01, ns indicates not significant.

Apart from the above interrelationships among traits, DBH was also observed to be significantly correlated with most leaf traits of the trees. With increasing DBH, the leaves of the individuals among canopy species exhibited significant increases in LT, leaf dry mass content, SD, PT, and leaf C and P contents. Conversely, SLA and SPL decreased (Figure 3). Meanwhile, understory species also showed significant increases in LT and PT with DBH size. These trends suggest a prevalent size‐dependent variation in the majority of leaf trait variables. Furthermore, canopy species, which have thicker PT, denser and smaller stomata, higher leaf dry mass content, and higher leaf carbon, nitrogen, and phosphorus content (Figure S3), exhibit stronger and more significant DBH‐trait correlations compared to understory species (Figure 3).

### 3.2 Trait‐Growth Relationships at the Individual Level

**FIGURE 4:**
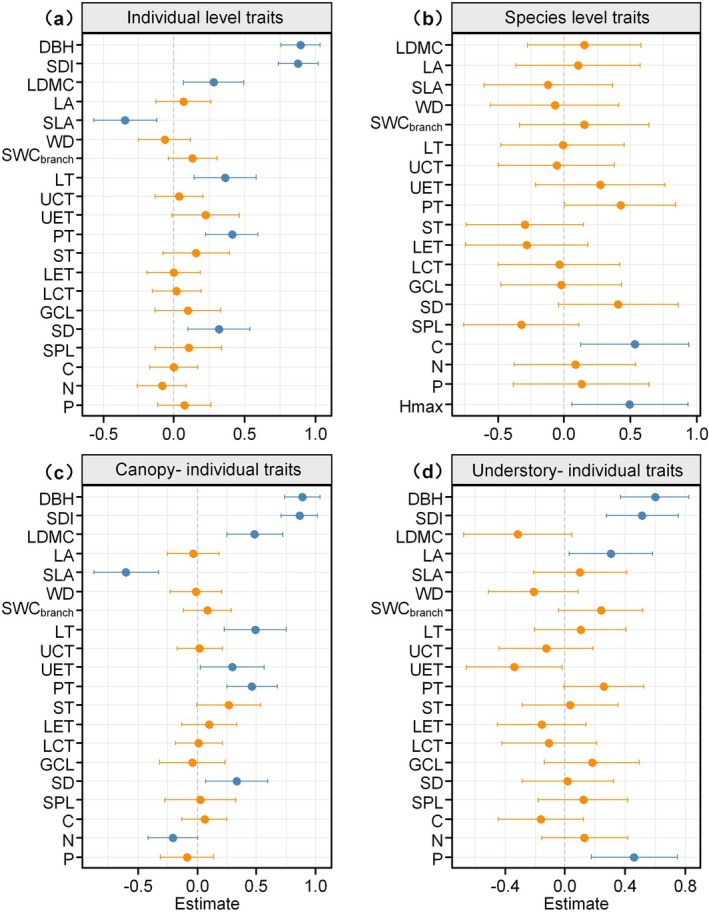
The effect of the different traits on annual growth of individual trees. Tree growth was explained by (a) traits at the individual level and by (b) average trait values at the species level. The impact of individual‐level traits is further explored for trees within canopy (c) and understory species (d), respectively. Each trait was independently evaluated as a fixed effect, with species treated as a random effect in the linear mixed model. The canopy group included both canopy and subcanopy species in the analysis. All explanatory variables were standardized. Blue points indicate the significant effect size, orange points indicate non‐significant effect size. C, leaf carbon content; DBH, tree diameter at breast height; GCL, guard cell length; LA, leaf area; LCT, lower cuticle thickness; LDMC, leaf dry matter content; LET, lower epidermis thickness; LT, leaf thickness; N, leaf nitrogen content; P, leaf phosphorus content; PT, palisade tissue thickness; SD, stomatal density; SDI, tree spatial dominance index; SLA, specific leaf area; SPL, stomatal pore length; ST, sponge tissue thickness; SWC_branch_, branch saturation water content; UCT, upper cuticle thickness; UET, upper epidermis thickness; WD, sapwood density.

Initial DBH and tree SDI were the most influential variables affecting the annual BAI (Figure 4, Figure S5). At the individual tree level, four traits—LDMC, LT, PT, and SD—were positively correlated with tree growth, while SLA had a negative impact (Figure 4a). These trait‐growth correlations also exhibited the same trend in the trait‐DBH relationships (Figure 3, Figure S2). In canopy tree species, LDMC, LT, UET, PT, and SD were positively correlated with BAI, while SLA and leaf N content had a negative impact (Figure 4c). In understory species, only LA and leaf P content showed significant and positive correlations with tree growth rate (Figure 4d). This indicates that the trait‐growth link is more prevalent in canopy species.

At the species level, only the average leaf carbon content (C) and maximum height significantly influenced BAI (Figure 4b), indicating that trait‐growth relationships are more prevalent when considering individual‐level trait values. Due to the contrasting effects of intra‐ and interspecific variations in leaf carbon content on tree growth, we further explored the linear relationships between leaf carbon content and growth, considering both the overall characteristics of the dataset and the specific characteristics of individual species. Figure S4 visually depicts the noticeable differences in the C‐growth relationships at both the overall and species‐specific levels. Specifically, leaf carbon content showed a generally positive correlation with growth across all individual data (*r* = 0.25, *p* < 0.05; Figure S4a). However, this positive effect observed was not driven by intraspecific variation in C but rather was attributed to interspecific variability. Only one canopy species (*Castanopsis eyrei* Tutcher.) showed a significant positive relationship between leaf intraspecific carbon content and individual growth rate (Figure S4). The interspecies trait‐growth relationship was further evidenced by the significant correlation between the average carbon content of species and BAI, as depicted in Figure S4b.

### 3.3 Trait‐Based Tree Growth Models

The stepwise regression results showed that the basic model involving initial DBH and SDI as predictors explained a significant portion of the variance in tree growth (BAI). The basic model had a marginal *R* ^2^ of 0.55 and a conditional *R* ^2^ of 0.62 (Table S1). Incorporating leaf PT and LT of individual trees into the basic model slightly improved the model fit ($R_{\text{fixed}}^{2}$ = 0.56, model 5), with leaf PT showing a statistically significant effect. Additionally, we examined the potential interaction effects of leaf traits with either DBH or spatial dominance in the basic model, but these interaction effects were not statistically significant.

For species‐level trait analysis, species *H*_max_ and C independently explained 10%, 11% of the variation in individual tree growth, respectively. However, including only leaf carbon content in the basic model led to an improvement in model fitting (Table S1). Utilizing C as the predictor for both the model intercept and slope of size dominance in the basic model yielded the best model‐fitting result in this study (ΔAIC = −13.64, $R_{\text{fixed}}^{2}$ = 0.60). By comparing the model fitting results of all the models (Table S1), we found that growth models based on individual‐level traits did not have an advantage in predicting tree growth compared to species average traits. In the optimal models for individual and species‐level traits (Model 4 and Model 8), the relative importance analysis (Figure S5) showed that BAI is mainly influenced by DBH and SDI, with a relatively lower sensitivity to other traits. The distribution of residuals for both models suggests that the variability in BAI not captured by the models is random, without showing systematic bias (Figure S6).

**FIGURE 5:**
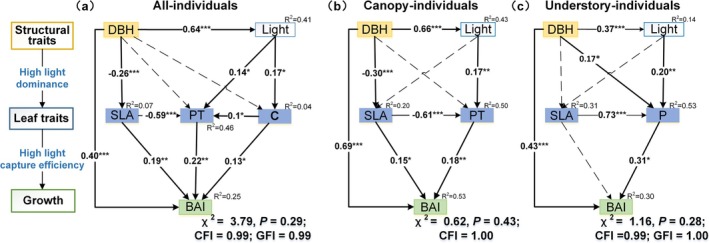
The path analysis performed for basal area growth rate. (a) illustrates the effects of individual traits on tree growth for all individuals; (b) illustrates how individual traits influence tree growth in canopy species; (c) shows the effects of individual traits on tree growth specifically in understory species. The canopy group includes both canopy and subcanopy species in the analysis. Variables in the diagrams are grouped into three categories, represented by distinct background colors—yellow for structural traits, light blue for leaf traits, and light green for growth process. Light means the tree crown illumination index. Solid lines represent significant paths, dashed lines represent nonsignificant paths, *p < 0.05, **p < 0.01, and ***p < 0.001.

The leaf traits‐based SEM (Figure 5) demonstrate that leaf traits had direct influences on BAI. DBH can also indirectly affect BAI through its impacts on leaf traits. Figure 5a shows that leaf PT was influenced by both SLA and leaf C content, indicating that changes in leaf light capture efficiency require a coordinated adjustment of carbon investment. Increases in both PT and leaf C content were positively correlated with light (crown illumination index). In the canopy species model (Figure 5b), SLA and PT demonstrate direct path coefficients of 0.15 and 0.18 with BAI, respectively. Both traits are directly affected by DBH and light condition, respectively, indicating that larger trees tend to have thicker PT and lower SLA. In the understory species model (Figure 5c), no significant relationships were observed between SLA and DBH. However, leaf P content showed a significant correlation with DBH, light, and SLA, and had a substantial positive path coefficient (0.31) with BAI, highlighting the important role of P content for the growth of understory species. The SEM models emphasize the combined significance of tree size structure and leaf functional traits in explaining tree growth.

## 4 Discussion

### 4.1 Individual Trait Variation and Its Size‐Dependent Effects

Most of the traits examined showed a high CV across all individuals, ranging from 7.52% to 68.55% across traits (Table 2), particularly notable in leaf structure‐related traits such as LA, SLA, and PT. The variation observed in the studied leaf traits is consistent with prior studies, which are closely linked to tree size and light exposure (Bin et al. 2022; Kenzo et al. 2022). However, we also found that variation in SLA was greater (42.09%) compared to previous studies (He et al. 2018; Poorter et al. 2018). This discrepancy can be primarily attributed to the extensive tree size range (5–61.8 cm in DBH) that covers diverse vertical environmental conditions, particularly the varying light conditions experienced by the leaves (Figure 5).

The effect of size‐dependent intraspecific trait variation (ITV) is recognized as a significant adaptive characteristic in complex‐structured natural forests (Kenzo et al. 2015; Bin et al. 2022; Zheng et al. 2022). Studies have demonstrated that this variation is strongly associated with environmental stress factors encountered at higher canopy levels, including elevated temperatures, intensified radiation, and stronger wind disturbances (McGregor et al. 2021; Bin et al. 2022). Based on our study, larger‐sized trees, which typically occupy dominant positions within their local surroundings, demonstrate specific adaptations in leaf functional traits such as increased PT and LDMC (Figures 3 and 5). Such changes can optimize photosynthetic capacity while reducing water transpiration by decreasing leaf size and SPL. In contrast, small understory trees in low‐light environments show conservative leaf economic investment with lower leaf N content, reflecting lower variation in morphological traits (LA, LT, PT, SD; Table 2), likely due to the relatively homogeneous understory environment (Sendall and Reich 2013; Cubino et al. 2021). These findings are consistent with the hypothesis that environmental heterogeneity is a crucial factor driving trait variation (Stark et al. 2017; Liang et al. 2019). In addition to environmental impacts, trait variation can also be attributed to the increased hydraulic path length in taller trees, which affects the structural coordination of leaf traits. This adaptation mitigates declines in water potential, thereby helping to maintain both photosynthesis and hydraulic homeostasis (Shiraki et al. 2017; Olson et al. 2020; Bauman et al. 2022). However, the lack of direct measurements of hydraulic traits in our study limits a comprehensive understanding of the co‐variation of hydraulic and photosynthetic traits. Future studies should focus on how the hydraulic and morphological traits of individuals are coordinated across varying vertical environmental gradients. It is also worth noting that taller trees typically access deeper soil water compared to smaller trees. This may lead to distinct hydrological niches and ontogenetic shifts in hydraulic strategies, which could further contribute to the greater trait variation observed in canopy species (Brum et al. 2023).

### 4.2 Correlations Between Functional Traits and Growth in Montane Cloud Forest

Our investigation demonstrates the individual‐level traits such as SLA, LT, and LDMC were significantly associated with growth (Figure 4a). However, these correlations are not evident when considering species‐level average trait values (Figure 4b). This highlights the importance of measuring individual‐level traits for a more direct understanding of tree demographic rates. On the other hand, we also identified a significant relationship between leaf carbon content and growth, which is attributed to interspecific variability rather than within‐species variations (Figure S4). Such discrepancy is not atypical or uncommon in trait‐based ecological studies. Previous studies have suggested that trait–growth relationships are not necessarily consistent within and across species, proposing that a species‐based approach might better capture the potential growth rates tied to the overall adaptive strategy of species (Poorter et al. 2018; Yang et al. 2021). In contrast, employing an individual‐level approach could more accurately reflect realized growth rates in response to actual environmental conditions (Poorter et al. 2018). Our findings support the notion that both individual and species perspectives yield unique insights in trait‐based ecological studies.

Previous studies have shown a generally positive SLA–growth relationship, which is predicted by the leaf economic spectrum and indicative of a resource acquisition strategy for rapid growth (Wright et al. 2004; Rawat et al. 2021). This pattern is commonly observed in tree saplings, both among and within species (Fajardo and Siefert 2018). However, when considering individuals of various sizes and ontogenetic stages, the commonly observed positive SLA–growth relationship across species can be contrary to the intraspecific SLA–growth relationship (Yang et al. 2021; Bauman et al. 2022). A potential explanation is that the relationship between any single trait and growth depends on the overall phenotypic context. For example, in vertically stratified forests, significant light‐dependent changes in SLA (1/LMA) contribute to the observed increase in net assimilation rate per area (Aa) (Keenan and Niinemets 2017). This high light‐driven trait plasticity leads to a general decrease in SLA values alongside modifications in other leaf anatomical traits, such as higher SD, shorter SPL, and thicker PT (Figure S2, Figure 5). Such structural adjustment results in enhanced water‐use efficiency and carbon assimilation (Kenzo et al. 2015; Brienen et al. 2017), which can further compensate for the potential decrease in total carbon assimilation associated with lower SLA. Furthermore, the negative relationship between SLA and growth among individuals is significantly influenced by tree size. Typically, larger trees, which tend to have smaller SLA, achieve accelerated growth rates due to their increased competitive advantages in resource acquisition (Gray et al. 2019; Bauman et al. 2022). In fact, incorporating both individual tree size and leaf traits into the structural equation model reveals a positive relationship between SLA and growth (Figure 5).

Our results demonstrate that canopy species adopt a fast acquisition strategy by increasing leaf PT and SD, which are associated with enhanced light capture efficiency. The thickening of PT not only increases nitrogen per unit LA but also expands the surface area of mesophyll cells, thereby reducing diffusion resistance and boosting photosynthetic capacity (Coble and Cavaleri 2017; Gonzalez‐Paleo and Ravetta 2018). In contrast, understory species adopt a conservative growth strategy suited to low light conditions with low SD and low leaf N content (Figure 5, Figure S3). Previous studies have shown that, compared to nitrogen, adjusting leaf P content is a vital strategy for understory species in subtropical forests, where availability of phosphorus is limited (Zhu et al. 2014; Liu et al. 2024). Our results confirmed that modulation of leaf P content is essential for dealing with low light conditions in understory species (Figure 5c). This may be because, in low‐light and phosphorus‐limited subtropical forests, increased leaf P content significantly improves CO_2_ assimilation rate of leaves under these conditions (Liu et al. 2017).

### 4.3 The Contribution of Leaf Functional Traits in Improving Conventional Individual Tree Growth Models

In line with our initial expectations, the regression results indicated that including individual traits as predictors had limited effects on improving the tree growth models. Among the 18 traits examined, only the PT emerged as a significant predictor in the multivariate model (Table S1), despite the significant correlations observed between other traits such as SLA, LT, SD, and BAI (Figure 4a). This indicates that the influence of individual tree traits on growth may be diluted when more general factors, such as tree size, are considered in the multivariate model. Variations in leaf traits of individual plants were influenced by DBH and the spatial dominance of trees (SDI). The results of SEM further confirmed that the trait‐growth relationships were mediated by tree size and light status (Figure 5). Moreover, results showed that the most effective growth model did not include individual‐level functional traits. Instead, it utilized species‐level leaf carbon content as a fixed effect, influencing both the model intercept and slope of the SDI (Table S1, model 8). While this improved model fit, a single species‐level measure of leaf carbon content provides limited insight into tree growth strategies or physiological processes. Additionally, the substantial intraspecific variation in leaf carbon content limits its practical utility in growth models due to the challenge of obtaining consistent and reliable estimations across individuals (Figure 2).

Our findings support the hypothesis that vertical light availability are key ecological drivers of trait variation, as documented in other montane forests. These vertical gradients shape distinct trait strategies across canopy layers, contributing to the size‐related variation observed in leaf structure and function. Accordingly, evaluating tree growth performance in cloud forests requires accounting for ontogenetic changes in trait expression, especially those linked to tree size and spatial dominance. While leaf functional traits provide meaningful insights into the physiological and ecological mechanisms of tree growth, their explanatory power is often modulated by structural attributes and local competitive context. Rather than focusing solely on leaf traits, future trait‐growth modeling efforts should consider the importance of individual tree size and spatial dominance. Overlooking these aspects could lead to an overemphasis on the role of functional traits in growth prediction. In addition, our study primarily focused on anatomical and phenotypic traits of trees, without delving into more nuanced physiological traits such as mesophyll conductance, stomatal conductance, and Rubisco efficiency. Investigating these physiological traits in future research could achieve a more comprehensive understanding of tree growth dynamics in cloud forests.

## 5 Conclusion

Our findings revealed significant size‐dependent individual trait variation patterns, indicating individual trait often covaries with tree size to jointly enhance physiological and morphological adaptations throughout ontogeny. Several traits associated with photosynthetic capacity, such as PT, SLA, and SD, were identified to significantly influence tree growth, suggesting the importance of leaf photosynthetic structure in affecting tree performance in the light‐limited montane cloud forest. As trees grow larger, canopy species enhance light capture ability by adjusting the morphological structure of their leaves, such as low SLA and thick palisade tissue, while understory species adapt by increasing leaf P content, reflecting specialized adaptations to their respective vertical niches. Furthermore, the study identified that tree growth was predominantly influenced by the size of trees and their spatial dominance over the surrounding neighbors, and due to the size‐related trait variation effects, it suggests that the potential utility of leaf traits in optimizing conventional tree growth models is limited. It is recommended to consider tree size and spatial dominance in future trait‐based studies for a comprehensive understanding of the relationship between functional traits and their ecological impacts.

## Author Contributions

**Yong‐Qiang Wang:** conceptualization (equal), formal analysis (equal), funding acquisition (supporting), investigation (equal), methodology (equal), writing – original draft (equal), writing – review and editing (equal). **Shi‐Dan Zhu:** data curation (equal), investigation (equal), writing – review and editing (equal). **Han Wang:** funding acquisition (supporting), writing – review and editing (equal). **Kun‐Fang Cao:** project administration (equal), supervision (equal), writing – original draft (equal). **Hong‐Xiang Wang:** conceptualization (equal), data curation (equal), formal analysis (equal), funding acquisition (lead), investigation (equal), methodology (equal), writing – original draft (equal), writing – review and editing (equal).

## Conflicts of Interest

The authors declare no conflicts of interest.

## Supporting information


**Figure S1:** Relationships between DBH (diameter at breast height) and tree height (a) and crown illumination index (b).
**Figure S2:** Bivariate (Pearson) correlation matrix among functional traits across 322 individual trees.
**Figure S3:** Differences in leaf traits between canopy layers.
**Figure S4:** Association between leaf carbon content and basal area increment (BAI) growth.
**Figure S5:** The relative importance of the fixed effect variables in models 4 and 8.
**Figure S6:** Distribution of residuals in regression models 4 and 8.
**Table S1:** The selected models for predicting individual tree annual basal area increment (BAI) and their statistical outputs.

**Data S1:** ece372169‐sup‐0002‐DataS1.xlsx.

## Data Availability

The data supporting the findings of this study have been uploaded as a Supporting Information Dataset with the submission.
