# Supplementary material for: Size‐Related Variation in Tree Leaf Traits and Its Effects on Trait‐Growth Relationships in a Subtropical Cloud Forest
Source: Ecol Evol. 2025 Sep 15;15(9):e72169. doi: 10.1002/ece3.72169 (PMC12435977; doi:10.1002/ece3.72169)

**Supplementary Information**

**Title:** Size-related variation in tree leaf traits and its effects on trait-based growth modeling in a subtropical cloud forest

**Authors:** Yong-Qiang Wang, Shi-Dan Zhu, Han Wang, Kun-Fang Cao, Hong-Xiang Wang

**Figure S1.** **Relationships between DBH (diameter at breast height) and tree height (a) and crown illumination index (b).** Height means tree height. Illumination index means tree crown illumination index, ranges from 1 to 5, where 1 signifies no direct light, 2 represents low lateral light, 3 shows some vertical light (with 10% - 90% of the vertical projection of the crown receiving vertical illumination), 4 means the crown is entirely exposed to vertical light but lateral light is obstructed within part or all of the 90% inverted cone surrounding the crown, and 5 indicates that the crown is completely exposed to both vertical and lateral light. DBH means the diameter at breast height of tree. Light blue for canopy tree species, orange for understory tree species.

**
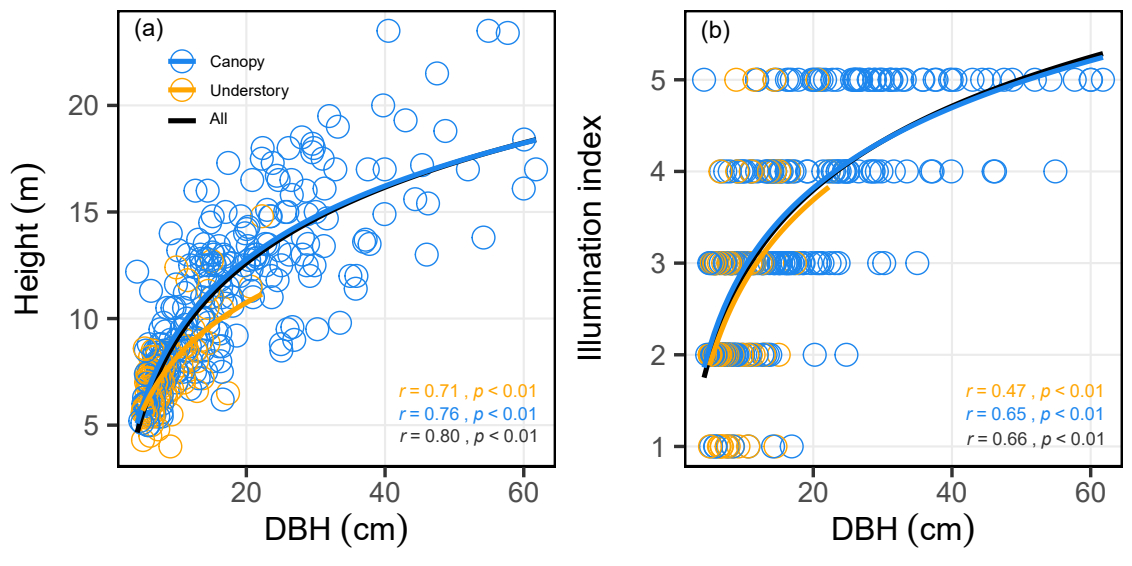
**

**Figure S2.** **Bivariate (Pearson) correlation matrix among functional traits across 322 individual trees.** Abbreviations: DBH (Tree diameter at breast height), SLA (Specific leaf area), WD (Sapwood density), SWC_branch_ (Branch saturation water content), LT (Leaf thickness), LA (Leaf area), LDMC (Leaf dry matter content), UCT (Upper cuticle thickness), UET (Upper epidermis thickness), PT (Palisade tissue thickness), ST (Sponge tissue thickness), LET (Lower epidermis thickness), LCT (Lower cuticle thickness), SD (Stomatal density), SPL (Stomatal pore length), GCL (Guard cell length), C (Leaf carbon content), N (Leaf nitrogen content), P (Leaf phosphorus content). (*) indicates *p* < 0.05, (**) indicates *p* < 0.01, and (***) indicates a significant level at *p* < 0.001.


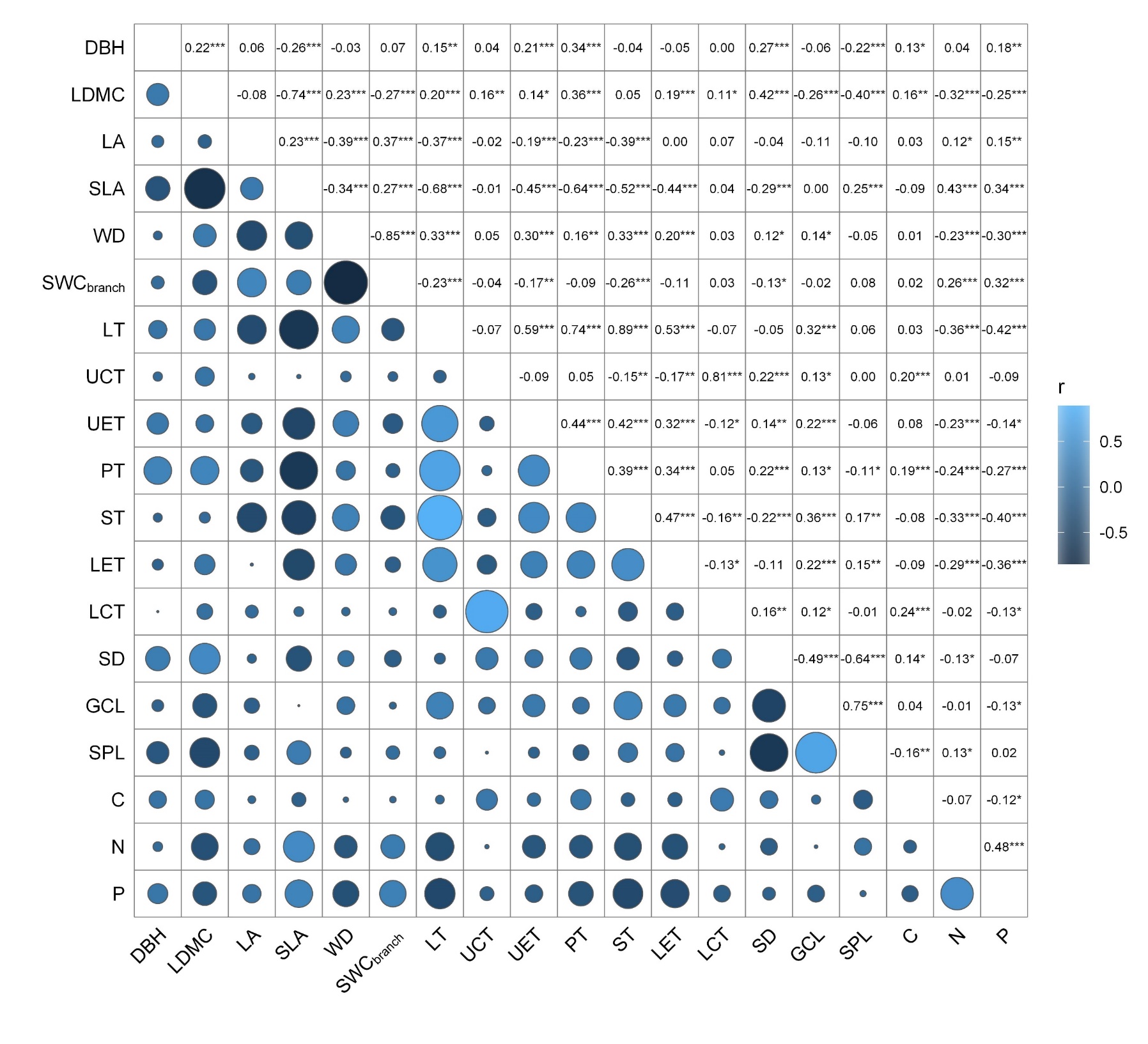


**Figure S3.** **Differences in leaf traits between canopy layers: (a) specific leaf area; (b) leaf dry matter content; (c) leaf thickness; (d) leaf palisade thickness; (e) stomatal density; (f) stomatal pore length, (g) leaf C content; (h) leaf N content; (j) leaf P content.** Box plots include the median value, the interquartile range, 95% confidence intervals, and outliers. Light blue for canopy tree species, orange for understory tree species. Statistical significance was determined by t-test: (***) *p* < 0.001; (**) *p* < 0.01; (*) *p* < 0.05; (NS) not significant.


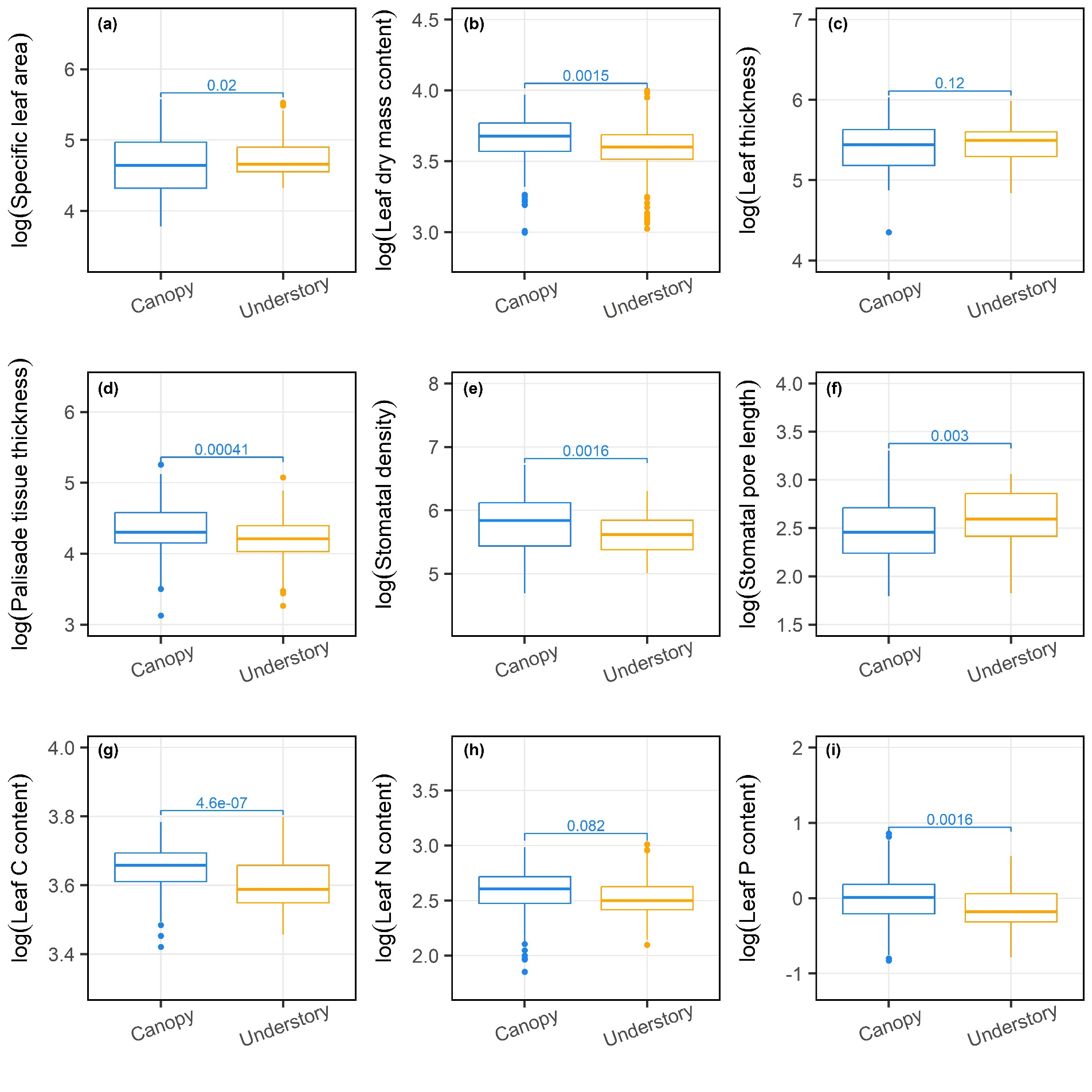


**Figure S4. Association between leaf carbon content, specific leaf area (SLA) and** **basal area increment (BAI).** (**a**) The correlation of individual tree leaf carbon content with BAI across various species. The bold red line indicates the aggregated trend for the entire dataset, while the multi-colored lines represent the species-specific correlations. (**b**) A scatterplot demonstrates the relationship between the average leaf carbon content and average BAI for each species. (**c**) and (**d**) show the same relationships as (**a**) and (**b**), but for specific leaf area (SLA). The shaded area indicates the 95% confidence interval. BAI_s_ refers to the mean basal area increment growth at the species level, and leaf carbon content_s_ and specific leaf area_s_ represent species-level mean values. Each point in (b) and (d) represents a species. (*) indicates a significant level at *p* < 0.05, ns indicates a non-significant level at *p* > 0.05.


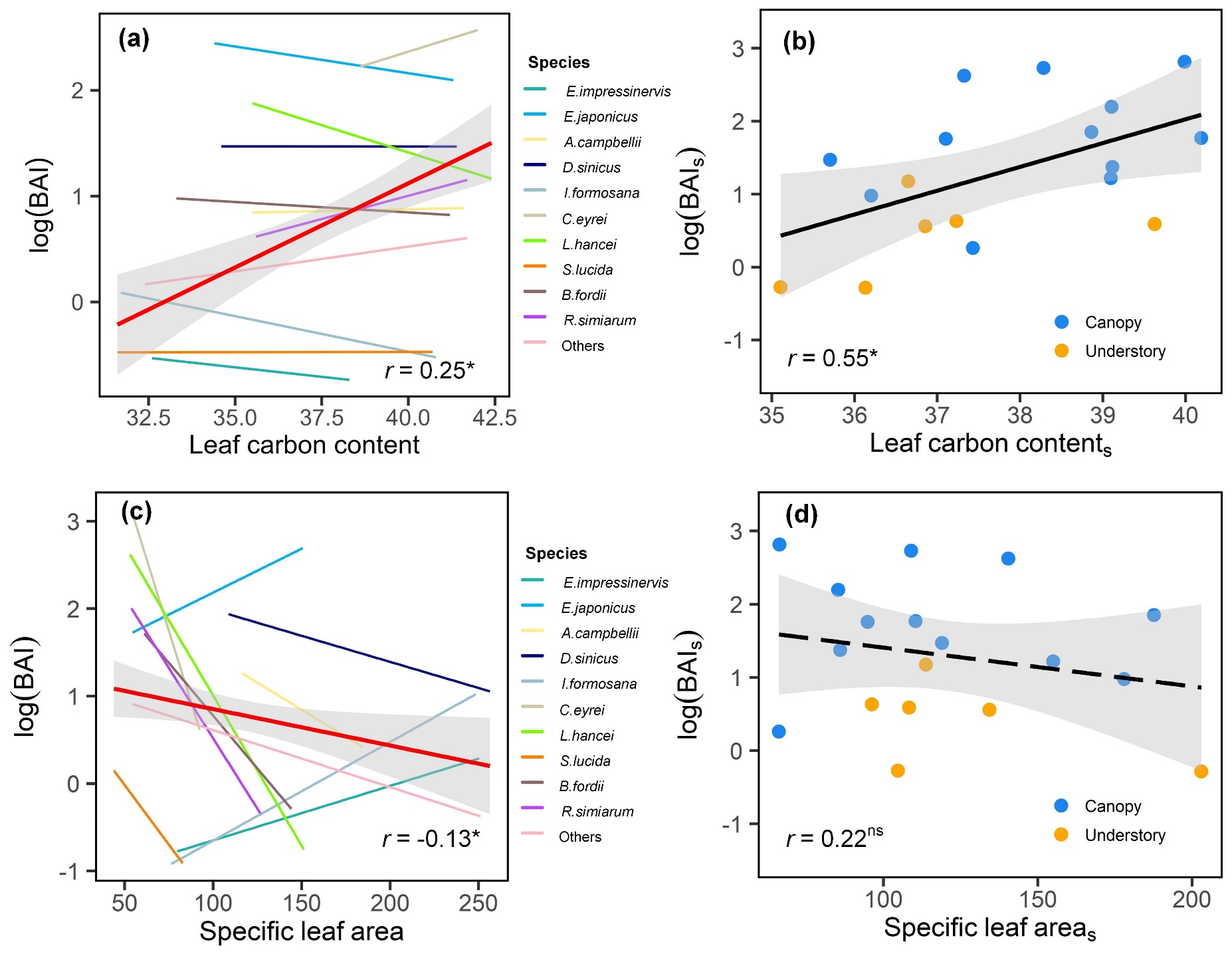


**Table S1.** **The selected models for predicting individual tree annual basal area increment (BAI) and their statistical outputs.**

| **Data type** | **Model** | | **AIC** | **ΔAIC** | **Marginal R^2^** | **Conditional R^2^** |
| --- | --- | --- | --- | --- | --- | --- |
| Individual-level traits | 1 | *β_0j_*+*β_1j_*DBH | 639.98 | 0 | 0.53 | 0.61 |
|  | 2 | *β_0j_*+*β_1j_*DBH +*β_3_*SDI | 634.16 | −5.82 | 0.55 | 0.62 |
|  | 3 | *β_0j_*+*β_1j_*DBH +*β_2_*SDI +*β_3_*PT | 633.39 | −6.59 | 0.55 | 0.62 |
|  | 4 | *β_0j_*+*β_1j_*DBH +*β_2_*SDI +*β_3_*PT+ *β_4_*LT | 632.13 | −7.85 | 0.56 | 0.63 |
|  | 5 | *β_0j_*+*β_1j_*DBH +*β_2_*SDI +*β_3_*PT +  *β_4_*PT*SDI+ *β_5_*LT | 633.80 | −6.18 | 0.56 | 0.63 |
| Species-level traits | 6 | *β_0j_* +*β_1j_*DBH +*β_2_*SDI+*β_3_*H_max_ | 635.43 | −4.55 | 0.55 | 0.62 |
|  | 7 | *β_0j_* +*β_1j_*DBH +*β_2_*SDI+*β_3_* C*_j_* | 629.22 | −10.76 | 0.59 | 0.64 |
|  | 8 | *β_0j_* +*β_1j_*DBH +*β_2j_*SDI  *β_0j=_γ_00_+γ_01_*C*_j_ + υ_j_*  *β_2j =_ γ_20_+γ_21_* C*_j_* | 626.52 | −13.46 | 0.60 | 0.63 |

Note: BAI and DBH were log-transformed in each model. SDI (Spatial Dominance Index) represents the hyperbolic tangent index that quantifies tree spatial dominance. PT refers to the leaf palisade tissue length of individual trees, LT denotes leaf thickness, C*_j_* is the leaf carbon content of species *j*, and H_max_ is the species maximum height, respectively. *β* and *γ* are model parameters, and *υ*_j_ was included as the random effect for species *j*. ΔAIC is the difference in the Akaike’s information criterion between a model and the model 1.

**Figure S5. The relative importance of the fixed effect variables in models 4 and 8**, which are the optimal models based on individual- and species- level functional traits, respectively.


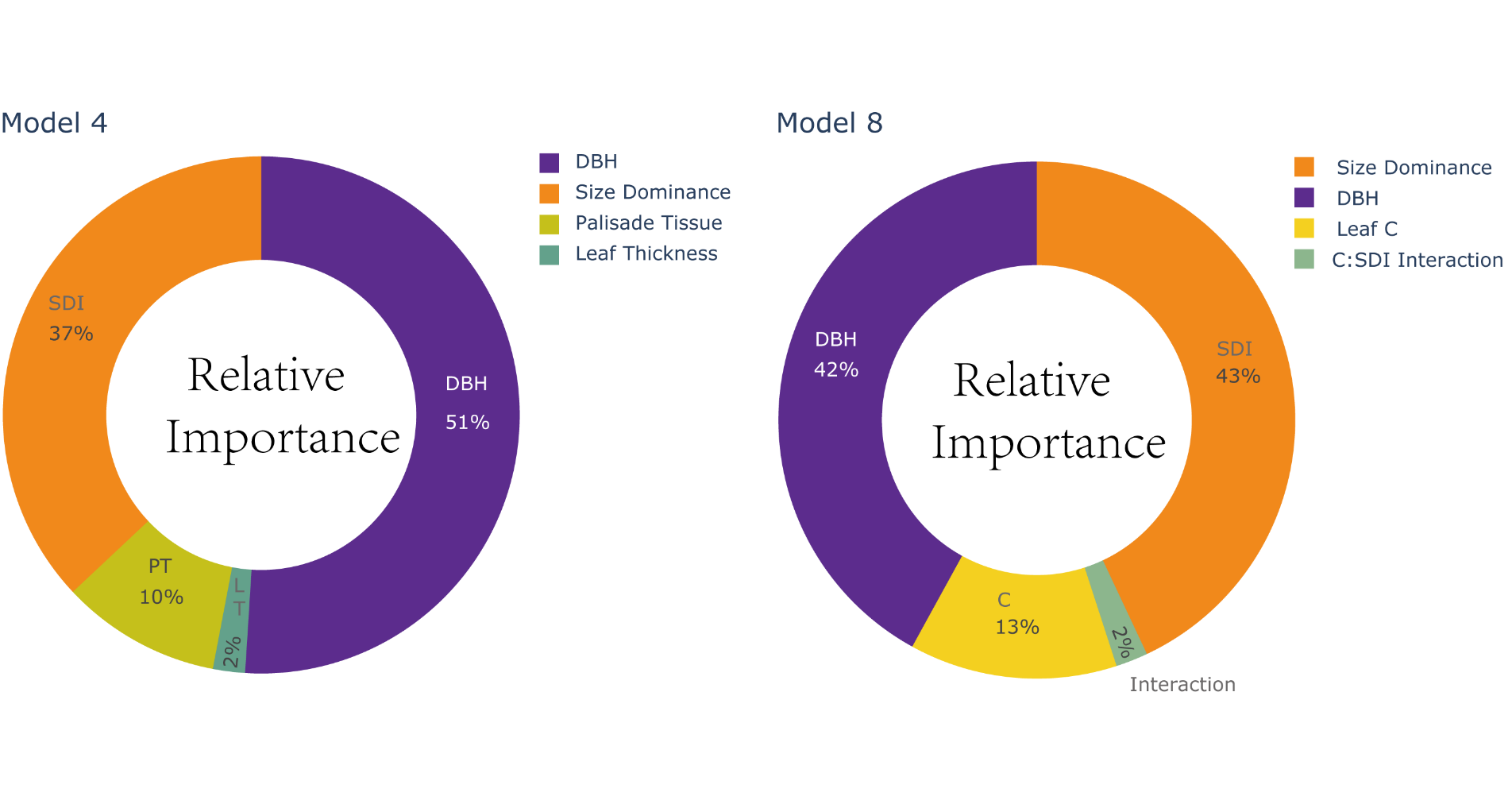


**Figure S6. Distribution of residuals in regression models 4 and 8.**


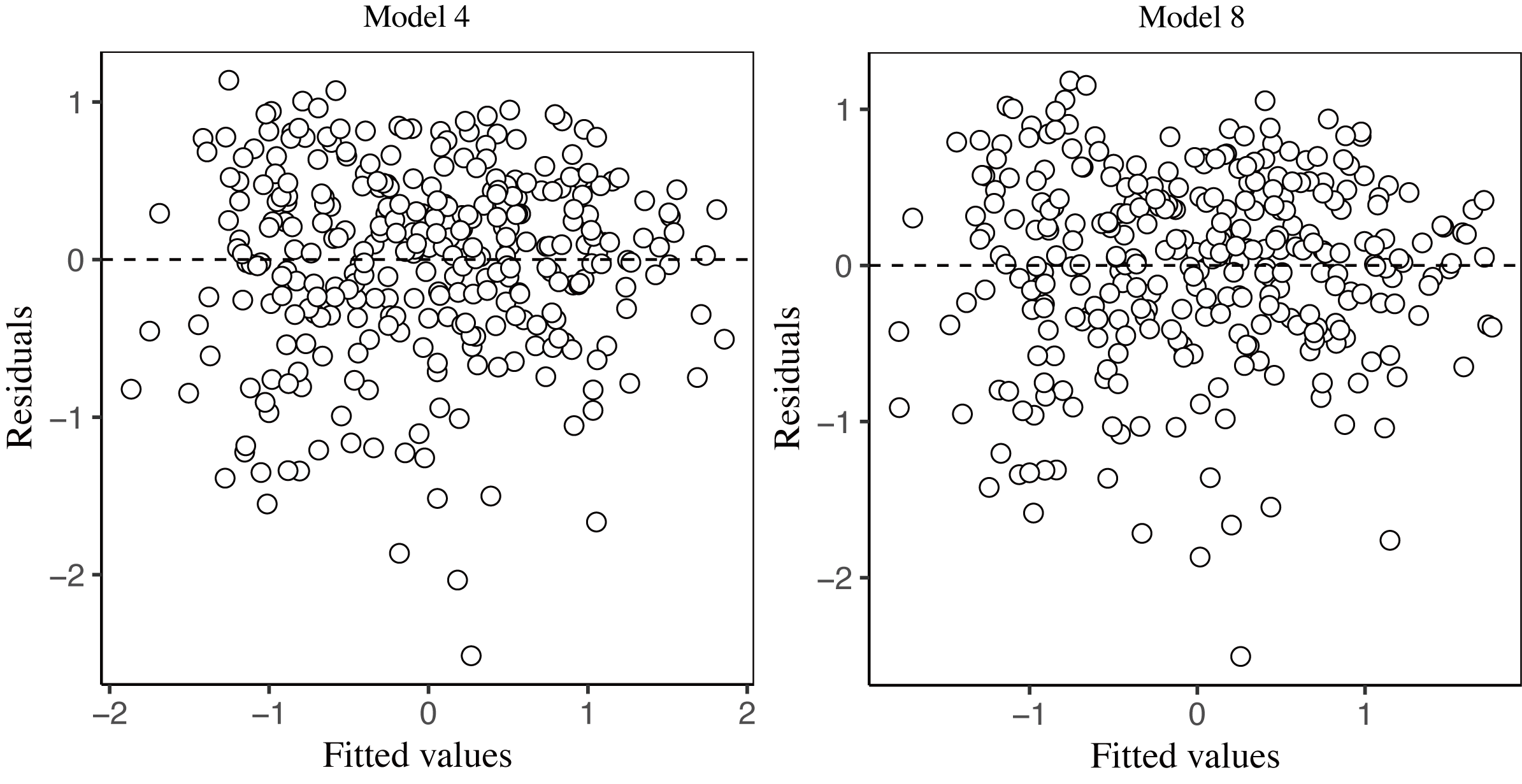

Supplement: Supplementary file 1 — Figure S1: Relationships between DBH (diameter at breast height) and tree height (a) and crown illumination index (b). Figure S2: Bivariate (Pearson) correlation matrix among functional traits across 322 individual trees. Figure S3: Differences in leaf traits between canopy layers. Figure S4: Association between leaf carbon content and basal area increment (BAI) growth. Figure S5: The relative importance of the fixed effect variables in models 4 and 8. Figure S6: Distribution of residuals in regression models 4 and 8. Table S1: The selected models for predicting individual tree annual basal area increment (BAI) and their statistical outputs. [file ECE3-15-e72169-s002.docx]
